# P-448. Antibiotic use and risk for Clostridioides difficile Enterocolitis in Children

**DOI:** 10.1093/ofid/ofaf695.663

**Published:** 2026-01-11

**Authors:** Lindsey Davidson, Deepa Mukundan, Shipra Singh, Mounika Polavarapu

**Affiliations:** University of Toledo College of Medicine and Life Sciences, Toledo, OH; University of Toledo College of Medicine and Life Sciences, Toledo, OH; University of Toledo College of Medicine and Life Sciences, Toledo, OH; University of Toledo College of Medicine and Life Sciences, Toledo, OH

## Abstract

**Background:**

Antibiotic use is one of the most important risk factors for *Clostridioides difficile* infection (CDI). Most research on the impact of antibiotic use and CDI has been done on adult populations. Several studies have found that the antibiotic class associated with the highest risk for CDI is clindamycin, followed by fluoroquinolones, and then the 3rd generation cephalosporins. The spectrum of antibiotics used in children is slightly different from that of adults, and hence antibiotics with highest risk for CDI could be different in children. Thus, our objective was to determine the antibiotic classes that are associated with the greatest risk for *Clostridioides difficile* enterocolitis (CDE) in children.

**Methods:**

This study used TrinetX which is a global network providing real world datasets that include over 113 million de-identified patients. We used ICD-10 codes to identify patients from ages 0-18 who were prescribed clindamycin, fluoroquinolones, 3rd generation cephalosporins, or penicillin. We then identified those who developed CDE for each of the classes of antibiotics stated above. We then compared the clindamycin cohort to each of the other cohorts for the incidence of CDE with p < 0.05 and an odds ratio over 1 considered as significant.

**Results:**

We found 5,897,721 patients who used one of the 4 antibiotics. Of those, we found a higher incidence of CDE among those who were prescribed clindamycin compared to the other three antibiotic classes. Specifically, we found that the odds ratio of CDE in the clindamycin cohort compared to fluoroquinolones, 3rd generation cephalosporins, and penicillin was 1.508, 1.17, and 3.091 respectively

**Conclusion:**

Pediatric patients taking clindamycin are at the greatest risk of developing CDE similar to adults. The increased risk associated with clindamycin was followed by 3rd generation cephalosporins, fluoroquinolones, and penicillins in that order. This is different from adult studies which found that fluoroquinolones hold the greatest risk following clindamycin for CDI.

**Disclosures:**

All Authors: No reported disclosures

